# Multilevel Upper Body Movement Control during Gait in Children with Cerebral Palsy

**DOI:** 10.1371/journal.pone.0151792

**Published:** 2016-03-21

**Authors:** Aurora Summa, Giuseppe Vannozzi, Elena Bergamini, Marco Iosa, Daniela Morelli, Aurelio Cappozzo

**Affiliations:** 1 Interuniversity Centre of Bioengineering of the Human Neuromusculoskeletal System, Department of Movement, Human and Health Sciences, University of Rome “Foro Italico”, Rome, Piazza Lauro De Bosis 15, 00135 Rome, Italy; 2 Fondazione Santa Lucia IRCCS, Via Ardeatina 306, 00179 Rome, Italy; Duke University, UNITED STATES

## Abstract

Upper body movements during walking provide information about balance control and gait stability. Typically developing (TD) children normally present a progressive decrease of accelerations from the pelvis to the head, whereas children with cerebral palsy (CP) exhibit a general increase of upper body accelerations. However, the literature describing how they are transmitted from the pelvis to the head is lacking. This study proposes a multilevel motion sensor approach to characterize upper body accelerations and how they propagate from pelvis to head in children with CP, comparing with their TD peers. Two age- and gender-matched groups of 20 children performed a 10m walking test at self-selected speed while wearing three magneto-inertial sensors located at pelvis, sternum, and head levels. The root mean square value of the accelerations at each level was computed in a local anatomical frame and its variation from lower to upper levels was described using attenuation coefficients. Between-group differences were assessed performing an ANCOVA, while the mutual dependence between acceleration components and the relationship between biomechanical parameters and typical clinical scores were investigated using Regression Analysis and Spearman’s Correlation, respectively (α = 0.05). New insights were obtained on how the CP group managed the transmission of accelerations through the upper body. Despite a significant reduction of the acceleration from pelvis to sternum, children with CP do not compensate for large accelerations, which are greater than in TD children. Furthermore, those with CP showed negative sternum-to-head attenuations, in agreement with the documented rigidity of the head-trunk system observed in this population. In addition, the estimated parameters proved to correlate with the scores used in daily clinical practice. The proposed multilevel approach was fruitful in highlighting CP-TD gait differences, supported the in-field quantitative gait assessment in children with CP and might prove beneficial to designing innovative intervention protocols based on pelvis stabilization.

## Introduction

Locomotion is the result of a number of complex interactions involving neuromuscular activity, joint movements, bone alignment, and the rules that govern bodies in motion [1]. Typically, the parameters investigated have been spatiotemporal parameters and lower limb joint mechanics characterizing physiological walking patterns. However, since a considerable portion of the human body mass is located above the pelvis, the scientific literature is increasingly considering the analysis of upper body motion. In this respect, empirical observation suggests that the trunk plays an important dynamic role in balance control and gait stability [2,3].

Gait stability has been referred to as the capacity to minimize oscillations during walking from the lower to the upper levels of the human body [4]. Acceleration data measured at different body levels in the three anatomical directions can provide insightful information about gait stability [5]. Using either Root Mean Square (RMS) values [6] or frequency domain measures [7], upper body accelerations have been described in healthy subjects. Specifically, healthy subjects typically present a progressive reduction of acceleration from pelvis to sternum and from sternum to head which reflects the adoption of postural control strategies. As a consequence, the head moves on a straight line at an almost constant speed during walking [8,9], leading to a steady visual input and more effective processing of the vestibular system signals, thus improving control of equilibrium [10]. In the case of any loss, or alteration, of physiological motor functions, as is the case of neurological disorders, the above mentioned control strategy can be defective, and consequently the physiological stabilization of the head may be compromised.

Cerebral palsy (CP) encompasses two-thirds of all childhood disabilities and refers to a group of permanent disorders, mainly related to movement and posture, attributed to non-progressive disturbances that occurred in the developing fetus or the neonatal brain [11]. The disruption of normal brain maturation can cause failure in acquiring an appropriate locomotor schema, or the emergence of atypical locomotor patterns usually related to an asymmetrical, slower and less stable gait compared to that of typically developing (TD) children [12,13]. The locomotor patterns exhibited by children with CP have been widely studied in terms of lower limb kinematics, using a classical gait analysis approach [14]. The control of upper body movements during gait has also been assessed in children with CP, either using motion analysis [15,16], or by adopting musculoskeletal models such as the foot placement estimator [17]. Increased ranges of motion in head and trunk movements have been observed in each anatomical plane during gait [18,19]. In addition, assessment of the upright gait stability in these children suggested that their trunk movements are the result of both compensatory movements due to lower limb impairments, and to a trunk control deficit [20]. Consequently, it can be hypothesized that children with CP could present problems in attenuating the existing high accelerations from lower to upper body.

Only recently, adopting a single inertial sensing unit attached to the lower back was proposed to carry out global assessments in children with CP [5,21]. The authors reported that children with CP encountered difficulties in dynamic balance control during gait compared to TD children, as suggested by wider oscillations of the trunk, increased accelerations at pelvis level and asymmetry in all three anatomical directions. However, at the present level of knowledge, the way subjects with CP manage accelerations from the pelvis to the head remains to be addressed.

The purpose of this work is to characterize how children with CP attenuate upper body accelerations from the pelvis to the sternum and the head, in comparison with a group of TD children. To this aim acceleration data were collected at the three anatomical levels with a multilevel approach based on the use of magneto-inertial sensors. The characterization of the upper body accelerations, and how they are transmitted through the trunk, may allow to gain specific insights about gait strategy and locomotor patterns in children with CP, thus supporting therapists in defining *ad hoc* rehabilitation programs aimed at the proper development and maintenance of walking ability in children.

## Methods

### Ethics statement

All participants gave their assent and either their parents or legal guardians provided written informed consent according to the declaration of Helsinki. The experimental protocols were approved by the Ethics Committee of the Santa Lucia Foundation.

### Participants

Twenty children with clinical diagnosis of CP and twenty TD children were recruited. Both groups were matched for gender (11 males, M, and 9 females, F) and age (CP: 5.70 ± 2.27 years, range 2–9 years, TD: 5.85 ± 2.18 years, range 2–9 years), and were characterized by similar stature (CP: 1.10 ± 0.18 m, TD: 1.19 ± 0.18 m). To take into account for the effect of knee flexion contractures during gait [22], effective leg length was also measured as the distance between the greater trochanter and the lateral malleolus while standing (CP: 0.54 ± 0.11 m, TD: 0.54 ± 0.09 m). The CP group included children with hemiplegia (15), diplegia (4), and dystonia (1). The whole group was classified by expert physiotherapists according to the Gross Motor Function Classification System (GMFCS) [23], as follows: 12 patients at GMFCS1 level, 6 at GMFCS2 level, and 2 at GMFCS3 level. Since the rehabilitation gym was an environment familiar to the children, with the floor free of any obstacle, the latter two patients (aged 6 and 9) were able to complete the experimental protocol without any hand-held assistive device, even if classified as GMFCS3. The CP group was assessed by the Gross-Motor-Functional-Measures-88 (GMFM) [24] and all the children were characterized by a GMFM score higher than 45. All TD subjects involved in the study were physically active and had no neurological, orthopaedic or motor disorder.

### Equipment and data acquisition

**Fig 1:**
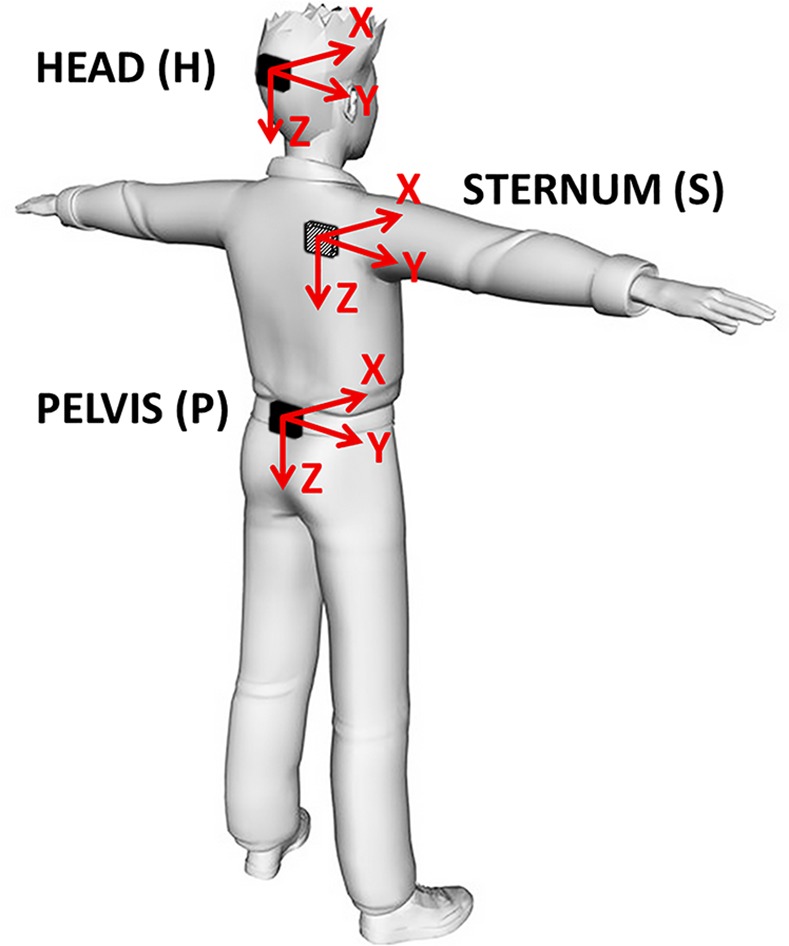
Sensor location and axes orientation of the Magneto-Inertial Measurement Units (MIMUs) attached on the children’s body segments. H: head level; S: sternum level; P: pelvis.

Three magneto-inertial measurement units (MIMUs) (Opal, APDM Inc., Oregon, USA) were used to collect 3D acceleration, angular velocity and magnetic field vector components using the Motion Studio software. Using the MIMU proprietary algorithm, the orientation of each unit was also obtained. Sample rate was set to 128 samples/s, and full-range scale was set to ± 2g (with *g* = 9.81 m/s^2^), ± 1500°/s and ± 600 μT, for the three sensors, respectively. Children were dressed with adjustable supports including swim caps, stretch tops and stretch shorts, with pockets tailored to house the MIMUs. The three units were positioned at head level (H), on the occipital cranium bone close to the lamboid suture, at sternum level (S), in correspondence to the center of the sternum body, and on the pelvis (P), at sacrum-L5 level (Fig 1). The three MIMU orthogonal axes were carefully aligned to the cranio-caudal (CC), medio-lateral (ML) and antero-posterior (AP) axes of each body segment, respectively. Consistently with the above-cited literature, and for the sake of readability, the term “upper-body” is used in the present work to refer to torso, head and pelvis body segments.

Children were asked to maintain a standing position for about 3 s and then to perform a 10-meter walking test on a linear pathway at their self-selected walking speed looking ahead. The start and stop lines of the pathway were marked by two visible stripes. Each subject performed three trials. Video recordings were collected for each trial using a commercial video-camera (JVC GC-PX10 HD memory) to control trial performance during data post-processing.

### Data analysis

Data were processed using custom algorithms implemented in Matlab^®^ (The MathWorks Inc., MA, US). The inclination of each MIMU with respect to gravity was first computed during the initial part of each test, when each participant was stationary in a standing position. Each MIMU reference frame was then rotated, through a rigid transformation, following the pitch-roll-yaw rotation sequence so as to have one axis aligned with gravity during the above-mentioned stationary phase. Then, for each body segment, a local anatomical frame was defined to match the rotated MIMU reference frame and was characterized by the CC, AP and ML axes. The gravitational acceleration component was removed from the measured acceleration signals using the orientation data provided by the MIMUs’ proprietary algorithm. The acceleration components, recorded during walking, were low-pass filtered using a 4^th^ order Butterworth filter. The cut-off frequency was determined by performing a residual analysis [25] on each acceleration component. The values obtained were similar for different components, trials, and participants (standard deviation less than 2 Hz). Thus, the cut-off frequency value was conservatively set to 20 Hz for all trials. The accelerations were then expressed in the relevant local anatomical frame.

The time taken to complete the 10-meter walking test and the foot striking events were obtained by identifying consistent features (maxima) on pelvis acceleration time histories using the methodology described in [26] and by comparing this information with video data. From this information, both step frequency (Number of steps/Time [s^-1^]) and average step length (Total distance/Number of steps [m]) were estimated.

Only the central part of the pathway was considered for further data processing. In particular, steady state walking phases were identified as in [27] and, for each trial, two consecutive strides (four steps) were selected within these phases and further considered for analysis to reduce the effect of walking speed variations on the AP acceleration measures. The average walking speed ([m·s^-1^]) was computed as the ratio between the average length of the two analysed strides and the relevant duration. To account for the influence of the participants’ stature on average step length, step frequency and walking speed, the corresponding dimensionless normalized parameters were computed as described in [28]. The average of the values obtained on the three tests was further considered for each subject.

The Root Mean Square of accelerations (RMS_a_) were then computed at the three levels of the upper body for each stride, using both the magnitude (Euclidean norm) of the acceleration as well as each component as expressed in the corresponding anatomical frame. The variation of the RMS_a_ from a level *i* to an upper level *j* of the upper body was assessed by computing attenuation coefficients (C_PS,_ C_SH,_ C_PH_) defined as follows:

$$C_{ij}=\left(1-\frac{{RMS}_{aj}}{{RMS}_{ai}}\right)\cdot 100$$

Average values of these parameters over different strides, trials, and participants were then obtained.

### Statistical analysis

The statistical analysis was performed using the IBM SPSS Statistics software package (IBM Corp., Armonk, NY, U.S.A.). The alpha level of significance was set to 0.05 for all statistical tests described hereafter. The D'Agostino & Pearson omnibus normality test was performed on all estimated parameters. Descriptive statistics and box plot analysis were performed on both RMS_a_ and attenuation coefficients. Using group and gender as factors, a two way analysis of covariance (2w-ANCOVA) adjusted for age was performed on normally distributed parameters to analyse between-group differences in both RMS_a_ values and attenuation coefficients, for each anatomical axis. Similarly, a rank analysis of covariance was performed using the Quade’s test in order to investigate the same aspect in non-normally distributed parameters.

Regression lines were obtained to investigate the following RMS_a_ inter-component relationship at the pelvis, sternum and head levels: RMS_a_ML *vs* RMS_a_CC, RMS_a_AP *vs* RMS_a_CC and RMS_a_AP *vs* RMS_a_ML. The quality of the regression fitting was quantified in terms of coefficients of determination (R^2^). To evaluate whether there was an interaction between groups and each covariate (one of the RMS_a_ components at a time), regression coefficients were compared using covariance analysis [29]. The analysis reported both TD and CP angular coefficients (mTD and mCP, respectively) of each regression line.

Finally, the Spearman’s rank correlation coefficient (ρ) was used to assess the relationship between each estimated parameter and the GMFM values. The same analysis was conducted to verify the presence of possible relationships between attenuation coefficients and the observed RMS_a_ values.

## Results

No significant differences was found between the TD and CP groups in either walking speed or normalized walking speed values. The RMS_a_ parameter was thus used for between-group comparisons [30]. The age-dependence of normalized walking speed was visually confirmed in the TD group (for details see S1 Fig, panel A), for which this parameter increased exponentially with age as in [31], whereas in children with CP this trend was not observed (for details see S1 Fig, panel B).

The results of the 2w-ANCOVA and of the Quade’s test for the main effect are presented in Table 1. Significant differences in spatiotemporal parameters were obtained only between the CP and TD groups, whereas no difference was found for the gender factor and for the group X gender interaction. Specifically, average step length was significantly smaller and step frequency significantly higher in the CP than in the TD group.

**Table 1:** Gait parameters obtained for both the TD and CP groups.

Parameter	TD	CP	F/U score	p
Walking speed (m/s)	0.91 ± 0.23	0.90 ± 0.23	0.020	0.889
Step length (m)	0.44 ± 0.10	0.39 ± 0.09	7.636	0.009 ^§§^
Step frequency (steps/s)	2.05 ± 0.23	2.31 ± 0.48	4.246	0.047 ^§^
Normalized walking speed	0.39 ± 0.08	0.40 ± 0.10	0.017	0.896
Normalized step length	0.81 ± 0.10	0.74 ± 0.10	3.848	0.058
Normalized step frequency	0.50 ± 0.09	0.52 ± 0.14	153.0	0.136

Descriptive statistics of parameters are expressed as mean ± standard deviation except of normalized step frequency reported as median ± interquartile range. F or U statistical scores are reported for the main effect. Significant differences (p < 0.05 or p < 0.01) are reported with the symbol § or §§, respectively.

The same analysis was initially performed on the magnitude of accelerations at pelvis, sternum and head levels. Again, only group-dependent differences were found (see Table 2). All the RMS_a_ values were significantly larger in the CP than in the TD group. RMS_a_ values were attenuated from pelvis to sternum and from sternum to head in the TD group, whereas in the CP group RMS_a_ values were attenuated from pelvis to sternum and amplified from sternum to head, indicating that attenuation between contiguous levels was different between groups. Specifically, C_PS_ was larger and C_SH_ was smaller in the CP than in the TD group, whereas C_PH_ was not statistically different between the two groups. In order to investigate if the larger C_PS_ observed in children with CP was associated with the larger pelvis RMS_a_, the correlation between these two parameters was computed, but it did not result statistically significant (ρ = 0.37, p = 0.105).

**Table 2:** RMS accelerations and attenuation coefficients in the TD and CP groups.

Parameter	TD	CP	F	p
Head RMS_a_ (m/s^2^)	1.24 ± 0.37	2.38 ± 1.08	17.757	< 0.001 ^§§^
Sternum RMS_a_ (m/s^2^)	1.33 ± 0.36	2.21 ± 1.25	8.043	0.008 ^§§^
Pelvis RMS_a_ (m/s^2^)	1.68 ± 0.43	3.50 ± 1.96	14.614	0.001 ^§§^
C_PH_ (%)	24.56 ± 16.88	24.32 ± 24.13	0.038	0.846
C_PS_ (%)	18.69 ± 14.16	33.30 ± 14.75	9.181	0.005 ^§§^
C_SH_ (%)	6.50 ± 12.90	-15.08 ± 24.16	12.708	0.001 ^§§^

RMS of the magnitude of the accelerations (RMS_a_) at pelvis, sternum and head levels and attenuation coefficients from pelvis to head (C_PH_), from pelvis to sternum (C_PS_) and from sternum to head (C_SH_) computed in the TD and CP groups. Results are expressed as mean ± standard deviation. Results of the 2w-ANCOVA for the group comparison are also reported and significant differences (p < 0.01) are indicated with the symbol §§.

**Fig 2:**
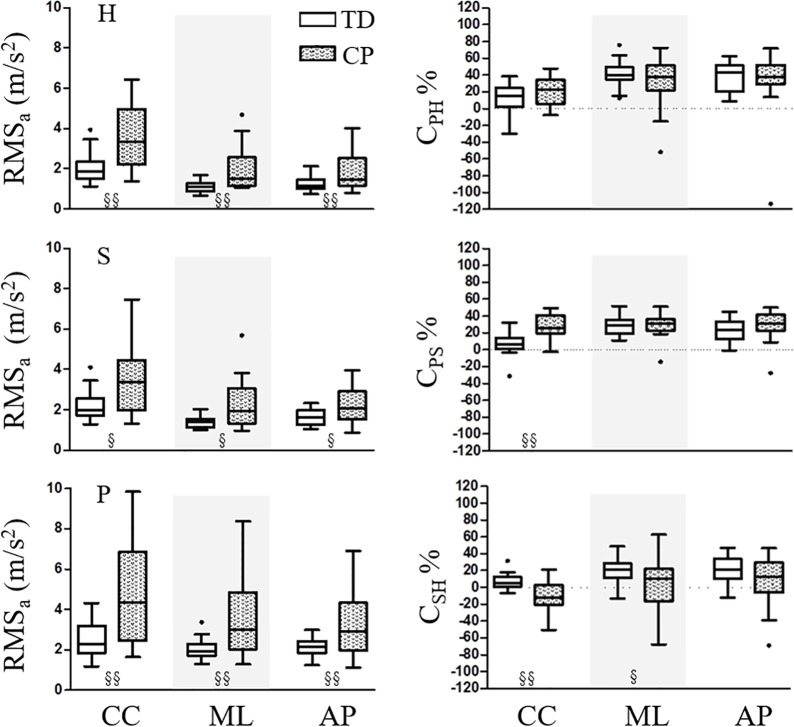
On the left, RMS of the acceleration components at the three levels. On the right, attenuation coefficients from pelvis to head (C_PH_), from pelvis to sternum (C_PS_), and from sternum to head (C_SH_) along the three anatomical axes. Parameters computed for the TD and CP groups are represented with empty and filled box-plots, respectively. Significant between-groups differences (p < 0.05 or p < 0.01) are reported with the symbol § or §§, respectively.

Furthermore, RMS_a_ parameters along CC, ML and AP directions are shown in Fig 2, with the relevant statistical analysis. RMS_a_ were significantly smaller in the TD than in the CP group (Fig 2A, left panel) and no dependence upon gender or group X gender was found. Concerning the attenuation coefficients, C_PS_ values in the CC direction were larger in the CP than in the TD group (p < 0.001), whereas C_SH_ in the CC and ML directions were found to be smaller (p < 0.001 and p = 0.019, respectively) in the CP than in the TD group (Fig 2B, right panel). As for the gender factor, a significant dependence was observed only for C_SH_ in the AP direction (TD: 5.66 ± 6.26% and 8.96 ± 11.83%, respectively for M and F; CP: -10.50 ± 19.06% and -12.22 ± 17.84%, respectively for M and F; p = 0.003). On the other hand, differences in the combined group X gender analysis were found for C_PS_ in the ML direction (TD: 25.25 ± 7.95% and 32.34 ± 14.05%, respectively for M and F; CP: 36.23 ± 10.45% and 22.34 ± 15.08%, respectively for M and F; p = 0.010).

**Fig 3:**
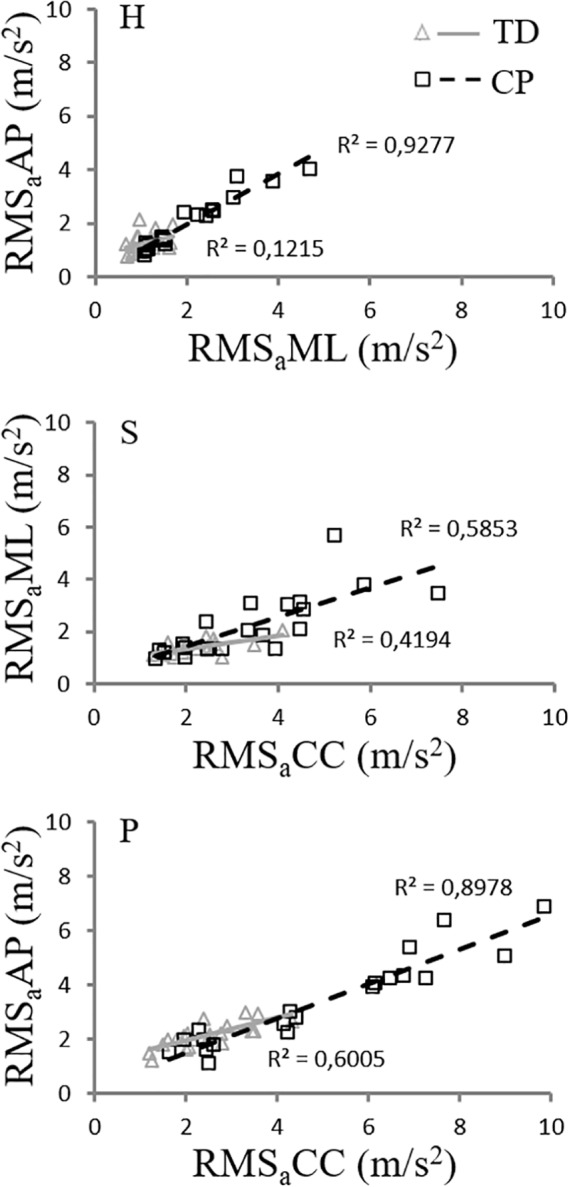
RMS_a_ inter-component relationships for the two groups (TD in light grey and CP in black). The inter-component comparison graphs characterized by significant different regression lines between the TD and CP groups are reported in figure: RMS_a_AP vs RMS_a_ML for head (p = 0.007), RMS_a_ML vs RMSaCC for sternum (p = 0.048), and RMS_a_AP vs RMSaCC for pelvis (p = 0.043).

Looking at the inter-component relationships at the different body levels, only three out of nine regression line pairs were significantly different between the two groups (p < 0.05; see Fig 3). Specifically, the following regression lines were significantly steeper in the CP than in the TD group: RMS_a_AP *vs* RMS_a_ML (mTD = 0.24, mCP = 0.96, p = 0.007) for the head, RMS_a_ML *vs* RMSaCC (mTD = 0.19, mCP = 0.54, p = 0.048) for the sternum, and RMS_a_AP *vs* RMSaCC (mTD = 0.28, mCP = 0.67, p = 0.015) for the pelvis.

For what concerns the correlation analysis performed between the estimated parameters and the patients’ clinical classification (GMFM), no significant correlation was identified either with the walking speed or with the normalized walking speed. Similarly, for the magnitude of the accelerations in the CP group, no significant correlation was found between the GMFM and RMS_a_ in any of the three levels. Conversely, focusing on the acceleration component, the GMFM negatively correlated with RMS_a_ML (ρ = -0.624, p = 0.004) and RMS_a_AP (ρ = -0.650, p = 0.003) at head level and positively correlated with C_PH_AP (ρ = 0.610, p = 0.006).

## Discussion

In the present study, a multilevel inertial sensor set-up was proposed to characterize segmental accelerations of pelvis, sternum and head during gait, and to investigate how these accelerations are attenuated from the bottom to the top in children with cerebral palsy (CP), when compared to age-matched children with typical development. Investigating accelerations and how they propagate through the upper body has a valuable physiological meaning and quantifies the role of upper body structures in reducing head accelerations: as a matter of facts, this role can be active, when looking at both trunk and arms movements during gait, as well as passive, when taking into account the contribution of the underlying anatomical structures (bones, muscles, tendons, cartilages).

The results about absolute and normalized gait velocities showed no statistical differences between the TD and CP groups, as already reported in previous studies [5,21]. It is worth noting that in the TD group, normalized walking speed exponentially increased with age according to the neuromaturation growth curve [31], whereas in children with CP this trend was not observed, probably due to the increased difficulties that children with CP might have in controlling longer and heavier body segments during growth [32]. For this reason, the effect of age differences among participants was taken into account by considering age as a covariate variable. Furthermore, dealing with other spatiotemporal parameters, average step length was significantly smaller and step frequency was significantly higher in the CP than in the TD group, in agreement with the existing literature [15].

The main contribution of the present study consists in the analysis of the the cranio-caudal (CC), medio-lateral (ML) and antero-posterior (AP) components of acceleration measured at pelvis, sternum, and head levels, which strongly confirmed the presence of greater acceleration values in the CP group at all three anatomical levels. These large accelerations may interfere with the normal processing of visual, vestibular, and somatosensory information regarding body position, thus inducing a vicious circle that potentially weakens the control of the upper body movement during gait, due to the altered sensory feedback. Consistently, it has recently been suggested that visuospatial deficits are crucial components of the disorder in spatial orientation, manipulation, locomotion, navigation, and even social interactions [33]. This impacts the motor strategy exhibited by children with CP: for instance, the forward pelvis and trunk tilts require that the head compensates by tilting backward to stabilize vision.

In the CP group, RMS_a_ values obtained at the lower trunk level were slightly higher than those previously reported [5,21], probably due to a pelvic sensor positioning at a lower lumbar level than in the cited studies. Analysis of the RMS of acceleration magnitude revealed that children with CP exhibited greater RMS_a_ values at each level than in the TD group, despite the comparable walking speed. This observation extends the previously known results about the pelvis. Higher RMS_a_ values at all upper body levels in the CP group are paralleled by increased dynamic ranges of motion in different planes observed in this population [18,19]. Previous studies about TD children reported attenuation of the accelerations from lower to upper body levels [34], highlighting gender differences similar to those observed in our data. However, similar considerations were not documented in children with CP.

This study confirms that the high RMS_a_ at pelvis level is an important clinical parameter justifying the reduced gait stability in the pathological group. It is revealed that even if patients affected by CP apply an increased pelvis-to-sternum attenuation, this does not adequately reduce high acceleration values at the pelvis level, exhibiting an RMS_a_ at sternum level that is still higher than in the TD counterpart. Specifically, in children with CP, C_PS_ is positive and significantly larger than in the TD group, thus indicating that children with CP attenuate more than TD children from pelvis to sternum. This circumstance could not be attributed to a deficit of the TD group, but potentially to the fact that for TD children, large attenuations are not strictly required to guarantee safe walking and an adequate dynamic stability of the trunk. Looking at the increased pelvis acceleration observed in children with CP, it is conceivable that a physiotherapeutic intervention to stabilize the pelvis may also reduce accelerations at higher body levels. However, the absence of a significant correlation between C_PS_ and RMS_a_ measured at the pelvis, probably due to the heterogeneity of the CP group that include both hemiplegic and diplegic patients, implies that the same percentage of reduction would be maintained also after that intervention. Regardless, the result will be a reduction of instabilities at sternum level.

Going upwards, the analysis of the sternum-to-head attenuation (C_SH_) presented a behaviour in the CP group that was the opposite to that of the TD group. While the latter group reduced accelerations from the sternum to the head, guaranteeing protection of the head, the former presented a negative C_SH_, meaning that acceleration increases from the sternum to the head. This inefficiency in attenuating these accelerations could be related to the documented rigidity of the head-trunk system in this pathology [20]. Summarizing, at head level children with CP do not reduce the high accelerations observed at pelvis level as TD children do (see Table 2). The observation that the RMS_a_ at sternum and head level maintain similar acceleration values, is consistent with the *en bloc* locomotion strategy adopted by children with CP to compensate for gait asymmetries that are typical of this population. In this respect, further research is needed to verify this interpretation, looking at similar patterns in other neuromuscular diseases or in amputee gait, as well as in a larger or more homogenous group of children with CP.

With respect to the different body axes, C_PS_ and C_SH_ along the vertical direction presented a significantly opposite trend between the two groups, allowing to associate to this direction the main differences in the control of upper body already observed in the magnitude acceleration. Consistently, the absence of differences in the attenuation of the accelerations along the direction of progression confirms the homogeneity of the two groups in terms of normalized spatiotemporal parameters (see Table 1), presenting similar effects when considering heel strike and foot progression on the AP axis.

The analysis of the inter-component relationship showed a specific behaviour of the CP group as compared with the TD group, highlighted by three significantly different linear trends (see Fig 3). It is observed that, as far as RMS_a_CC increases, children with CP report significantly steeper slopes for RMS_a_AP at the pelvis level and for RMS_a_ML at the sternum level. Similarly, as far as RMS_a_ML increases, children with CP present larger RMS_a_AP values at the head level. These increases in RMS_a_ values on the transversal plane, referred to the CC component, are in the order of 2.5 and 4 times larger than in TD children, for the AP and ML directions, respectively. This evidence suggests that the pathology may cause a loss of ability in disassociating the movement on different anatomical planes. While TD children are able to keep it in the sagittal plane, children with CP show a marked tendency to present movements that are coupled between planes. This is again in agreement with the above-mentioned documented rigidity of the head-trunk system [20] that entails higher accelerations at higher body level than in the TD child, considerably amplifying the effects of increased pelvis accelerations at upper body levels. These accelerations are mainly due to the walking pattern typical of children with CP, characterized by a succession of unstable steps and a sort of continuous effort to keep the centre of mass projection inside the base of support [35].

The estimated parameters showed high correlations with the GMFM clinical score. Specifically, as the GMFM decreases (i.e. as the severity of the pathology increases), RMS_a_ML and RMS_a_AP at the head level increase whereas C_PH_ in the AP direction decreases. Interestingly, these results enhance the potential clinical impact of these quantitative parameters as objective tools for the assessment of child instability.

In summary, children with CP did not show an adequate upper body stabilization as occurs in TD children, despite good attenuations from the pelvis to the sternum level. The existing thorax movements in children with CP during gait are the result of intertwining trunk control deficits and compensatory strategies due to lower limb impairments [20]. It is worth noting that the highly accelerated pelvis movement can be identified as the main factor inducing this modified walking pattern. Therefore, it is the authors’ opinion that neuro-rehabilitation treatment and intervention should focus more on stabilizing the pelvis, aiming to reduce these high accelerations. To do this, and based on the preliminary data obtained in the present work, more extensive studies are required to investigate the specific behaviour of subgroups divided according to the gravity of the pathology (GMFCS levels), the affected side (hemiplegia versus diplegia), as well as the gender or age.

## Conclusions

The present work focused on the quantitative assessment of upper body accelerations during gait and of their attenuation from the pelvis to the head, in children affected by cerebral palsy using wearable magneto-inertial sensors. The availability and accessibility of the inertial measurement equipment, in fact, does not limit its everyday use by clinicians. Moreover, characterizing how head stabilization is managed during gait in children with CP can be potentially useful to study gait patterns, including compensatory ones. In this respect, the potential role of trunk movements for compensating asymmetries in lower limb patterns was highlighted, specifically involving decreasing/increasing accelerations at the different body levels. The adopted biomechanical parameters were fruitful in highlighting gait differences between children with CP and their TD peers and can be used to support therapists and physicians either to drive the design of innovative intervention protocols or to prescribe *ad hoc* orthopaedic devices, and to monitor their efficacy in terms of gait stability.

## Supporting Information

S1 FigThe age-dependence of normalized walking speed.panel A) The age-dependence of normalized walking speed in children with typical development (TD). The normalized walking speed was defined as the product of the dimensionless step length and the dimensionless step frequency. According to the literature [31], an exponential neuromaturation growth curve can be described by the equation $y\left(x\right)=a\;\left[1-e^{-\frac{x}{b}}\right]$, with a = 0.4727, b = 36.233, R^2^ = 0.5927. panel B) Scattered plot for the age-dependence of normalized walking speed in children with Cerebral Palsy (CP). It is observed that the aforementioned neuromaturation curve disappears, having no trends that can fit the scattered CP group data.(TIF)
