# Supplementary figures and images for: Multilevel Upper Body Movement Control during Gait in Children with Cerebral Palsy
Source: PLoS One. 2016 Mar 21;11(3):e0151792. doi: 10.1371/journal.pone.0151792 (PMC4801392; doi:10.1371/journal.pone.0151792)

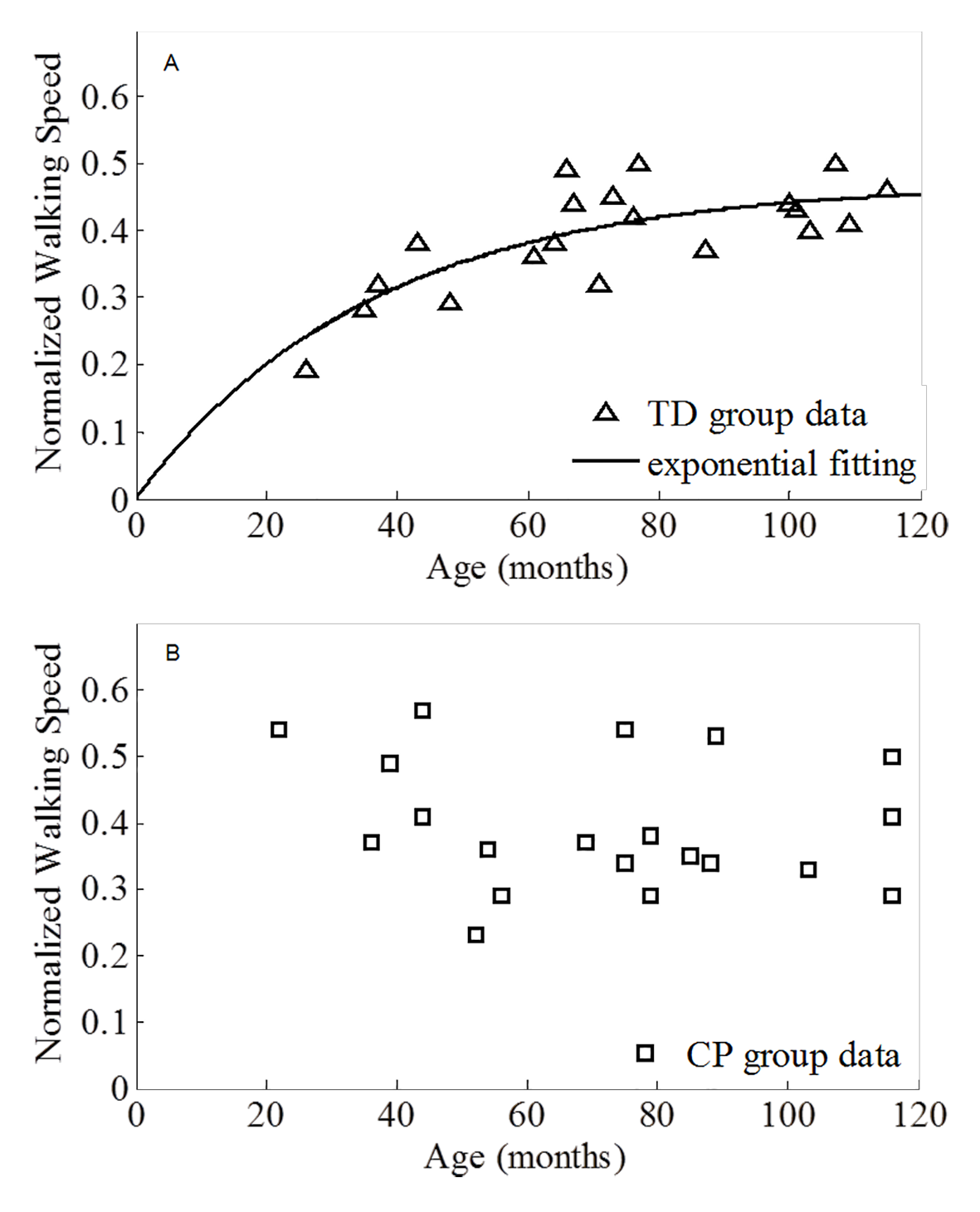

Supplement: S1 Fig — panel A) The age-dependence of normalized walking speed in children with typical development (TD). The normalized walking speed was defined as the product of the dimensionless step length and the dimensionless step frequency. According to the literature [31], an exponential neuromaturation growth curve can be described by the equation y(x)=a[1−e−xb], with a = 0.4727, b = 36.233, R2 = 0.5927. panel B) Scattered plot for the age-dependence of normalized walking speed in children with Cerebral Palsy (CP). It is observed that the aforementioned neuromaturation curve disappears, having no trends that can fit the scattered CP group data. (TIF) [file pone.0151792.s001.tif]
